# Human αβ and γδ T Cells in Skin Immunity and Disease

**DOI:** 10.3389/fimmu.2018.01304

**Published:** 2018-06-06

**Authors:** Michelle S. Cruz, Alani Diamond, Astrid Russell, Julie Marie Jameson

**Affiliations:** Department of Biological Sciences, California State University of San Marcos, San Marcos, CA, United States

**Keywords:** human, γδ T cell, skin immunity, T cell, diabetes, alopecia areata, melanoma, psoriasis

## Abstract

γδ T lymphocytes maintain skin homeostasis by balancing keratinocyte differentiation and proliferation with the destruction of infected or malignant cells. An imbalance in skin-resident T cell function can aggravate skin-related autoimmune diseases, impede tumor eradication, or disrupt proper wound healing. Much of the published work on human skin T cells attributes T cell function in the skin to αβ T cells, while γδ T cells are an often overlooked participant. This review details the roles played by both αβ and γδ T cells in healthy human skin and then focuses on their roles in skin diseases, such as psoriasis and alopecia areata. Understanding the contribution of skin-resident and skin-infiltrating T cell populations and cross-talk with other immune cells is leading to the development of novel therapeutics for patients. However, there is still much to be learned in order to effectively modulate T cell function and maintain healthy skin homeostasis.

## Skin as an Immunological Barrier

The skin serves as the largest organ in the body and as such provides a barrier against pathogens and regulates physiological changes. This is achieved through the network of cells, extracellular matrix molecules, and accessory organs residing in the complex layers of the skin. Human skin is composed of two compartments that include the epidermis and the dermis. The epidermis is a multilayer barrier composed of differentiating keratinocytes while the dermis is a connective tissue rich in collagen fibers (1). Immune cells including αβ and γδ T cells, and Langerhans cells reside in the epidermis. The dermis hosts a more diverse population of immune cells including: αβ and γδ T cells, dermal dendritic cells (DCs), innate lymphoid cells, plasmacytoid DCs, natural killer T cells, macrophages, mast cells, B cells, and fibroblasts (1). Human skin is estimated to host over 20 billion T cells or one million T cells per square centimeter (2). These T cells are composed of 1–10% γδ T cells with αβ T cells making up the remaining population (2, 3). Together, they mediate processes, such as skin homeostasis, wound repair, and immunity (4–6).

Skin-resident T cells recognize and respond to infected, stressed, or damaged cells by secreting cytokines and growth factors that stimulate cellular proliferation, induce cytolysis, and/or activate other cells to infiltrate the affected region (7–10). While T cells reside in both the epidermis and the dermis, the majority of T cells in normal human skin reside in the dermal–epidermal junction, in appendages, and near blood vessels (2). The epidermal and dermal compartments contain unique phenotypes of resident versus recirculating T cells with the epidermis exhibiting a higher frequency of CD103^+^ T resident CD4^+^ and CD8^+^ populations (11). CD103 along with β7 integrin bind E-cadherin which is expressed heavily by keratinocytes in the epidermis. Recirculating T central memory cells isolated from the skin express CCR7 and L-selectin, while T migratory memory lack L-selectin (11). Together these receptors provide signals that allow distinct populations of T cells to remain in the skin or recirculate to the blood or lymphatics during normal skin homeostasis.

## αβ and γδ T Cell Development and Migration to the Skin

Much of the work to understand T cell development has been performed using mouse models. Epidermal γδ T cells in mice, also known as dendritic epidermal T cells (DETC), seed the epidermis during fetal development in a wave and express a canonical Vγ3Vδ1 TCR (12, 13). It is unclear whether a similar seeding event occurs in humans; however, the majority of skin-resident T cells express the Vδ1 TCR (14, 15). It is important to note that in this review we employ the γδ TCR nomenclature described by Garman et al. (16) and Hayday et al. (17).

Chemokine receptors, cytokines, and adhesion molecules play key roles in T cell homing to the skin. In mice, CCR10 is upregulated on developing γδ T cells in the fetal thymus and required for efficient homing to seed the epidermis (18). In addition, CCR10 is utilized for T cell homing to inflamed skin *via* CCL27 produced by keratinocytes (19). In humans, vitamin D induces T cells to express CCR10 which may play a role in skin retention (20). T cells isolated from human skin also express the chemokine receptor, CCR8. The ligand for CCR8, CCL1, is expressed in the epidermis further suggesting that keratinocytes participate in T cell entry and retention in the skin through the production of chemokines (21). In addition to skin-resident T cells, circulating T cells home to a variety of barrier tissues upon infection and remain there poised for immediate effector functions to protect the organism (22, 23). The CCR6–CCL20 receptor ligand pair plays key roles in activated γδ T cell recruitment to the skin in mice (24). Skin-resident γδ T cells express CCR6, while the ligand, CCL20, can be expressed by keratinocytes, DCs, and endothelial cells. Human epidermal samples normally express low levels of CCL20; however, it is upregulated after an acute injury (25). Thus, CCL20 may act as an indicator of acute injury, initiating recruitment of infiltrating T cells to the epidermis.

The absence of cytokines, such as IL-7, IL-15, and IL-4, in mice results in a reduction/elimination of γδ T cells while IL-10 increases the generation of γδ T cells when present at low concentrations (26–29). These cytokines induce T cell survival and/or proliferation. IL-7R signaling induces rearrangement and transcription of the TCR γ-chain, while IL-15 facilitates γδ epidermal T cell precursor expansion and survival, and IL-4 signaling promotes growth of epidermal γδ T cells (30–33). Thus, critical roles are played by cytokine receptor signaling in γδ T cell development and expansion in sites such as the skin.

Selective recruitment of lymphocytes into human skin is facilitated by the expression of adhesion molecules on the T lymphocytes to ligands in the skin. For example, cutaneous lymphocyte antigen-1 expressed on a subset of human peripheral blood T cells, binds to E-selectin expressed by endothelial cells during inflammation (34). Endothelial cells express other adhesion molecules, such as intercellular adhesion molecule 1 (ICAM-1) and vascular cell adhesion protein 1, which also aid in T cell recruitment (35–37). In addition, the integrin CD103 is involved in the recruitment of T cells to the skin and binding to E-cadherin on epidermal cells (38–40). While CD103 is expressed in less than 15% of splenic T cells in mice and less than 3% of T cells in human peripheral blood, it is expressed at much higher rate on murine and human T cells in epithelial tissues (41–43). In mice, CD103 plays key roles in the establishment of γδ epidermal T cell populations as CD103-deficient mice show a significant reduction of γδ epidermal T cells and an impairment in morphology compared to controls (44). Together these chemokine receptors, cytokines and adhesion molecules develop/maintain skin-resident T cell populations and further recruit T cells to sites of inflammation in the skin.

## αβ and γδ T Cell Activation in the Skin

αβ T cell activation and cytokine production rely on three consecutive signals: TCR ligation, stimulation of costimulatory molecules and cytokine signaling (45–47). These three signals are essential for full functionality of the cell and without proper signaling there is a lack of T cell function, differentiation, proliferation, and survival (48). Co-stimulation is generated through the interaction between costimulatory molecules such as CD28 on the αβ T cell and ligands, such as CD80 and CD86 (46). γδ T cell activation is less understood; however, there are some similarities and differences with αβ T cell activation.

While αβ TCRs rely on MHC presentation of foreign peptides, γδ TCRs recognize some antigens in a manner that is more similar to antibody–antigen interactions (49). The entire repertoire of antigens recognized by γδ T cells is still unknown, yet it is clear that the γδ TCR is required for antigen recognition and the nature of antigen recognition is unique to the TCR expressed by the γδ T cell (49–51). The restricted TCR repertoire of Vγ and Vδ gene segments in both humans and mice leads to speculation that these TCRs recognize conserved self-proteins that become upregulated during stress, damage, or malignancy (52). Human γδ T cells are limited to Vδ1, Vδ2, and Vδ3 expressing populations which are distributed in different locations in the body (Table 1). γδ T cell populations have shown the ability to recognize atypical antigens, such as phosphoantigens, stress molecules including MHC class I related chain A molecules and MHC class I related chain B molecule, non-peptide metabolites of isoprenoid biosynthesis, and other unique antigens (53–55). One particular population of Vδ1^+^ T cells has been shown to recognize CD1 molecules with lipid antigens that are presented by antigen-presenting cells such as DCs (56). Specifically, CD1d is a Vδ1^+^ T cell ligand in both mice and humans (56, 57). In addition, γδ T cells expressing the Vγ2Vδ2 TCR have the ability to recognize phosphoantigens, which are important products of microbes, such as *Mycobacterium tuberculosis* (58). This suggests that antigen recognition between γδ T cells varies among populations and is likely unique to the site, such as the skin, in which they reside.

**Table 1:** γδ T cell subsets in humans and their roles in immunopathology.

			Features in immunopathology			
Subset	Location of highest prevalence	Identified antigen	Cancer	Psoriasis	Diabetes	Reference
Vδ1	Barrier tissues	MHC class I related chain A molecule/B, CD1 molecules, sulfatides	Immunosuppressive/regulatory roles Migrate to tumor site *via* CCR5 and CCR2 Produce IL-17, TNF-α, and IFNγ Kill melanoma cells			(53, 56, 59–63)

Vδ2	Peripheral blood	ULPB4, Phosphoantigens, F1-ATPase, aminoacyl tRNA synthetase	Migrate to tumor site *via* CCR5 and CXCR 3 Express adhesion molecules: lymphocyte function-associated antigen 1, L-selectin, CD44v6	Reduction in circulating CLA+ Vδ2 T cells Migrate to skin Produce TNF-α, IFNγ, IL-17A, and growth factors	Reduction in circulating Vγ2Vδ2 T cells in patients with high BMI Reduced IFN-γ production	(62–66)

Vδ3 (Vδ1−/Vδ2−)	Peripheral blood, liver	CD1d, CD1c	Increased number in patients with B cell chronic lymphocytic leukemia			(56, 67, 68)

While co-stimulation is less understood in γδ T cells, CD27 is expressed on most Vγ2Vδ2 T cells and contributes to T cell activation (69). Upon activation, the majority of CD27^+^ Vγ2Vδ2 T cells produce IFN-γ while IL-17 is rarely produced (69). CD27 signaling also protects against activation induced cell death and increases the expansion of tumor-specific cytotoxic T lymphocytes suggesting a costimulatory role (69). Studies suggest CD2 and ICAM-1 work as costimulatory receptors on Vδ1^+^ T cells (70). CD2 acts as a cell adhesion and co-stimulatory molecule that binds to CD58, facilitating cell contact and TCR ligation (71). ICAM-1 binds to lymphocyte function-associated antigen 1 inducing intercellular communication and inflammatory responses (72). In mice, the junctional adhesion molecule-like (JAML) is a costimulatory receptor for γδ epidermal T cell activation (73). Resting epidermal T cells express JAML at low levels; however, upon stimulation JAML expression is increased (73). Upon co-stimulation through JAML, epidermal T cells proliferate and produce IL-2, TNFα, and IFNγ (73). In addition another costimulatory receptor, CD100, regulates γδ epidermal T cell responses to keratinocyte damage (74). Ligation of CD100 facilitates the activated “round” morphology of epidermal γδ T cells through ERK kinase and cofilin (74). It will be important to determine whether these costimulatory pathways are also utilized by human skin γδ T cells.

## Skin-Resident T Cell Homeostasis and Epidermal Maintenance

Antigen-specific T cells expand during a skin infection and then largely die off leaving a small population of memory cells (11, 75). Recently, these remaining memory T cells have been categorized based on phenotype and function in humans (11). Skin-resident populations include dermal CD4^+^ CD103^−^ T cells and epidermal CD8^+^ or CD4^+^ T cells that express CD103. The epidermal T cells are less able to proliferate, but exhibit a greater ability to produce IFN-γ and TNF-α (11). Recirculating populations express CCR7 and lack CD69 but breakdown into L-selectin+ central memory cells and L-selectin- migratory memory cells (11). The T migratory memory populations are associated with expanding lesions in patients with cutaneous T cell lymphoma (11).

Memory CD4^+^, CCR10^+^, CCR6^+^, CCR4^+^ T cells homing to the skin can secrete cytokines that include IL-22, IL-26, and IL-23, which are involved in skin homeostasis (76). IL-22 acts on non-hematopoietic tissue cells of barrier tissues such as keratinocytes and helps regulate cellular differentiation and survival. This suggests IL-22 is involved in maintaining homeostasis of the epithelia (77). IL-23 induces the production of IL-22 and at elevated levels it contributes to a disruption in keratinocyte homeostasis (78). IL-26 has direct and indirect antiviral and antimicrobial properties, yet when not tightly regulated, skin homeostasis can become disrupted causing chronic infections and skin-related diseases (79). It is important to note that in most of these studies the T cell populations were not divided into αβ TCR versus γδ TCR expressing T cells. Thus, future studies will be needed to assess which subset includes the cytokine producing T cells that may be targets for therapeutic interventions.

In the murine epidermis, antigen-specific skin-resident memory CD8^+^ T cells and DETC adopt a dendritic morphology, increasing the number of cells they contact (75). DETC are distinctive in that they form phosphotyrosine-rich aggregates that keep the cells in a preactivated state (80). This dendritic morphology seems to be particular to the epidermis where receptors such as CD103 and E-cadherin interact, as dermal γδ T cells appear more rounded and are more motile (80, 81). At steady state, epidermal γδ T cells help maintain keratinocyte homeostasis through the production of factors such as insulin-like growth factor-1 (IGF-1) and wound healing through the production of keratinocyte growth factors (82–84). Mice lacking γδ T cells, TCRδ^−/−^ mice, exhibit delayed wound repair and fewer basal keratinocytes with increased differentiation (84, 85). However, when DETC are added to either the same well as TCRδ^−/−^ skin organ cultures or open wounds of TCRδ^−/−^ mice there is improved wound closure (84, 86).

Human skin-resident T cells have been speculated to also maintain skin homeostasis (5, 87). Both human and murine αβ and γδ skin-resident T cells regulate keratinocytes through the production of IGF-1; demonstrating their ability to influence keratinocyte proliferation and homeostasis (7, 82, 83). Recent work has focused on characterizing skin-resident T cells as compared to CLA^+^ T cells from blood (88). Results from this study confirm that CLA^+^ memory cells represent 80–90% of CD3^+^ cells in the skin and 15% in the blood (88). More interestingly, skin-derived T cells and blood-derived T cells express a different set of genes which are conserved in both mice and humans; these genes are involved in tissue homing and cell activation (88). Some gene signatures were consistent for T cells such as the cell markers *CTLA4, CD8A*, and *CD4* (88). T cells residing in human skin express higher levels of a variety of genes including *NR4A2* as compared to T cells in the blood which have elevated expression of genes such as *S1PR1* (88). Thus, T cells in the skin are transcriptionally unique from blood T cells and are comparable to previous gene signatures for T-resident memory cells.

## T Cells have a Variety of Functions in the Skin

In human skin, T cells have been shown to perform roles that maintain skin integrity. CD4^+^ αβ T cells such as Tregs and T-helper cells secrete cytokines in response to infection, tissue damage, and tumors (89). Th1 cells primarily secrete IFN-γ and IL-12 in response to intracellular pathogens that disrupt the skin barrier while Th2 cells fight extracellular pathogens and are involved in atopic diseases by secreting IL-4, IL-13, IL-24, IL-25, and IL-3 (90, 91). CD8^+^ T cells destroy infected cells by recognizing epitopes of viruses such as the herpes simplex virus, varicella zoster virus, and Epstein–Barr virus (92, 93). While most αβ T cells undergo apoptosis after a pathogen has cleared, a population of αβ T cells become long-lived memory T cells and reside in the skin (94, 95). These memory T cells are involved in inflammation upon viral infection by secreting cytokines, such as IL-22, IL-26, and IL-23 (76). While much of the published research in humans has focused on αβ T cell function in the skin, γδ T cells play key roles in maintaining skin integrity and protecting from malignancy.

γδ T cells monitor skin integrity by recognizing damaged cells and producing IGF-1 (7). Activated skin-resident T cells improve the rate of wound closure in cultured human skin in an IGF-1-dependent manner (7). In addition, human skin-derived γδ T cell clones exert cytotoxic responses against melanoma cell lines (96). Human dermal γδ T cells express the NKG2D receptor, which stimulates cell lysis (97, 98). Once activated, skin-derived γδ T cells produce perforin and induce Fas-mediated cytotoxicity (87). In patients with chronic lymphocytic leukemia of B-cell type, there is an increase in circulating Vδ1^+^ γδ T cells that respond to autologous leukemic B cells by proliferating and secreting TNF-α and IFN-γ (59). Although T cells play a vital role in providing an effective immune response, they can be harmful if not functioning in a regulated manner.

An imbalance in the number and/or function of skin-resident αβ and γδ T cells has been associated with chronic inflammation and skin-related diseases (99–102). Elevated skin-resident T cells has been reported in individuals with psoriasis and alopecia areata (99, 101). Alternatively, a reduction in T cell infiltration and function has been shown in individuals with type 2 diabetes and melanoma (100, 102). This has highlighted T cells as a promising target for immunotherapeutics. Here, we describe the current findings on T cells in human skin diseases and identify how they are being targeted with immunotherapeutics.

## Psoriasis

2–3% of the US population suffers from psoriasis, which is a chronic inflammatory skin disease (103). It is a multifaceted disease that can occur at any age, has unpredictable onset and remission, and is most commonly characterized by the presence of painful itchy skin lesions (104). Comorbidities of psoriasis include psoriatic arthritis, diabetes, ulcerative colitis, and cardiovascular disease (105, 106). While the mechanism of disease remains unclear, roles for innate immune cells and adaptive immune cells have been identified and participate in the pathogenesis.

**Figure 1:**
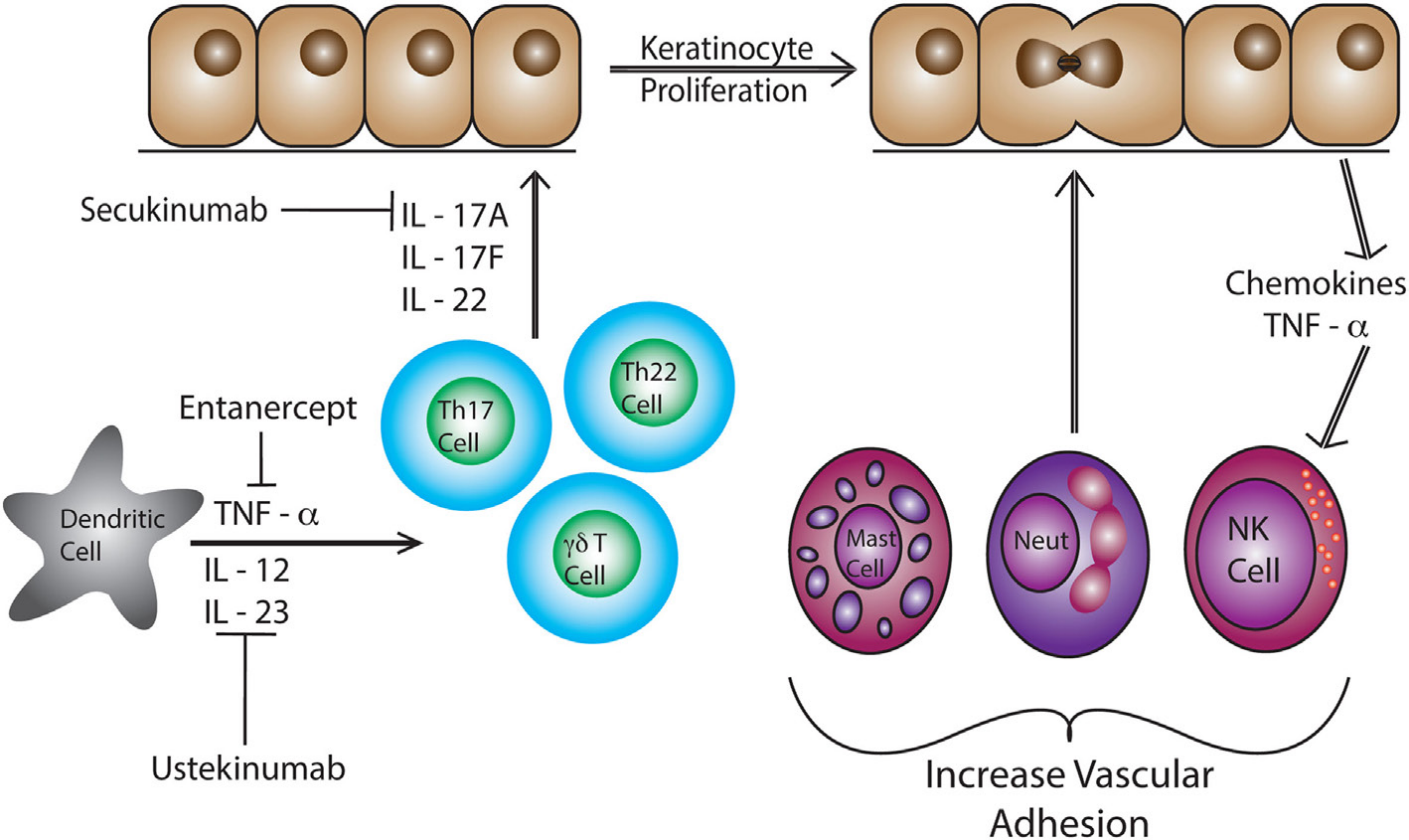
Dendritic cells produce IL-12, IL-23, and TNF- α in response to pathogen-associated molecular pattern activation. These pro-inflammatory cytokines induce differentiation of naïve T cells into Th17 and Th22 cells. These T cells produce IL-22, IL-17A, and IL-17F causing epidermal hyperplasia and induce epidermal chemokine and inflammatory cytokine production. Neutrophils, T cells, mast cells, and NK cells are recruited to the skin and then to the epidermal/dermal junction *via* changes in adhesion molecule expression, such as VLA-1 (CD49a).

Psoriatic lesions are caused by cross-talk between different cell types such as DCs and T cells, along with a number of cytokines including IL-17, IL-12, IFN-γ, TNF-α, and IL-23 (Figure 1) (107). Studies show an elevation in IL-23 and IL-12 production by macrophages and DCs in patients with psoriasis (108). Abnormal regulation of the IL-23/IL-17 axis in psoriasis has directed the focus of recent studies on infiltrating CD4^+^ and/or CD8^+^ T cells and skin-resident T cells, as contributors to disease pathogenesis (108–110). Dermal T cells are elevated in psoriatic skin compared to healthy skin, increasing from 1% CD3^+^ T cells to 15% in psoriatic samples (99). The proportion of dermal γδ T cells are also increased with more than 40% of the CD3+ T cells expressing the γδ TCR as compared to 15% in the healthy controls (99). Dermal αβ and γδ T cells also have the ability to secrete IL-17 when stimulated with IL-23, inducing inflammatory cytokines (63, 99, 111). In fact, dermal γδ T cells isolated from psoriasis patients produced more IL-17 upon IL-23 stimulation (99). These cytokines lead to the recruitment of more lymphocytes, neutrophils, and myeloid cells creating a positive feedback loop that maintains cutaneous inflammation and causes epidermal hyperplasia (112). Patients with psoriasis have a reduction in circulating CLA^+^ Vγ2Vδ2 T cells as compared to healthy patients (63). CLA^+^ Vγ2Vδ2 T cells are able to home to the skin and are elevated in number in the skin of patients with psoriasis (63). The CDR3 region of TCR genes was recently examined in both psoriatic and healthy patients. Interestingly, skin that has resolved psoriatic lesions retained IL-17-producing pathogenic oligoclonal αβ T cell populations, suggesting a mechanism by which disease can reoccur at the same site (113).

Anti-TNF-α therapies such as etanercept are effective at reducing inflammation and resolving psoriasis. However, the understanding of immune mechanisms and the pathogenesis of psoriasis is steadily progressing, allowing for more effective and targeted treatments such as ustekinumab and secukinumab (114). Ustekinumab, a human IgG1k monoclonal antibody, was approved by the FDA in 2009 to treat psoriasis in adults by neutralizing IL-12 and IL-23 (115, 116). Ustekinumab targets IL-12 and IL-23, specifically at the p40 subunit, preventing binding to the IL12Rβ1 and IL-23 receptor complexes on the surface of T cells and NK cells (117). Psoriatic patients treated with ustekinumab show a reduced expression of pro-inflammatory cytokine genes such as cyclin dependent kinase inhibitor 2D, IL-12B, and IL-17A (Table 2) (117, 118). IL-17 has been identified as a clinical target for the treatment of psoriasis. Secukinumab binds and blocks the activation of IL-17A which inhibits the production of β-defensin 2 and CXCL8 in human keratinocytes increasing pro-inflammatory cytokine production (119, 120). Elevated levels of IL-17 are a hallmark of psoriasis, therefore, rendering IL-17A inactive reduces inflammation (121, 122). Most recently, the FDA approved brodalumab. This monoclonal antibody binds and blocks the IL-17 receptor A (IL-17A) (123). The inhibition of IL-17R helps regulate and suppress inflammation mediated by IL-17 making it an additional target to treat psoriasis (119). While successful immunotherapies have been developed to treat the pro-inflammatory immune response associated with psoriasis, no drugs have been approved that cure the disease.

**Table 2:** Genes associated with T cell function and activation in patients with alopecia areata, psoriasis, diabetes mellitus type 2 or melanoma.

Region	Gene	Function	Involved in disease	Type of study	Reference
2q33.2	*CTLA-4*	Co-stimulatory family	Alopecia areata	Genome-wide association studies (GWAS)	(124)
4q27	*IL-21/IL-2*	T cell proliferation	Alopecia areata, psoriasis, diabetes mellitus type 2 (T2DM)	GWAS, Microarray	(124, 125)
9q31.1	*STX17*	Premature hair graying	Alopecia areata	GWAS	(124)
10p15.1	*IL-2RA*	T cell proliferation	Alopecia areata	GWAS	(124)
12q13	*ERBB3*	Epidermal growth factor receptor	Alopecia areata	GWAS	(124)
6p21.32	*MICA*	NKG2D activating ligand	Alopecia areata, psoriasis, T2DM	GWAS, microarray	(124, 125)
6p21.32	*HLA-DQA1*	Antigen presentation	Alopecia areata, psoriasis, melanoma	GWAS, microarray	(124, 126)
	*HLA-DRA*	Antigen presentation	Alopecia areata, melanoma	GWAS, microarray	(124, 126)
1p12	*NOTCH2*	T cell activation	T2DM	GWAS	(127)
8p21.3	*TNFRSF10A*	TNF receptor superfamily	T2DM	Microarray	(125)
	*CTLA2A*	Cytotoxic T lymphocyte-associated protein alpha	T2DM	Microarray	(125)
21q22.3	*ICOSLG*	T cell costimulator ligand	T2DM	Microarray	(125)
16Q12.1	*IL-4R*	T cell differentiation	Melanoma	Microarray	(126)

## Alopecia Areata

Alopecia areata is a polygenic autoimmune disease with a lifetime risk of 1.7% (128). Pathogenesis of alopecia areata involves the dysregulation of immune privilege around the anagen hair follicle (129). While the epithelial bulb of a normal anagen hair follicle does not express MHC Class I or MHC Class II; patients with alopecia areata exhibit increased MHC expression and adhesion molecule upregulation (130, 131). Elevated numbers of Th1 cells in the skin of alopecia patients produce IFN-γ which induces the expression of MHC class I molecules and triggers perifollicular CD8^+^ T cell infiltration (101, 132, 133). The severity of alopecia areata is closely related to CD8^+^ T cell gene expression, with a positive correlation between CD8^+^ T cell-specific genes and alopecia areata severity (101). Autoreactive CD8^+^ αβ T cells cause hair cycle arrest which then inhibits further hair growth (134).

Hallmark Th1 and Th2 cytokines are elevated in the blood of patients suffering from alopecia areata, while regulatory cytokines such as TGF-β are reduced (135). Patients with alopecia areata express a higher level of IL-23, IL-16, and IL-32 in the skin, which implicates the IL-17 inflammatory axis (136). Th17 cells are elevated in the scalp lesions of patients with alopecia areata, while FOXP3^+^ T regulatory cells are reduced (137). Alopecia areata lesions also exhibit upregulated expression of genes, such as *CCL19, IL-2, IL-15/IL-15RA, IL-2RA/IL-2RB*, and *Janus kinase 3* (*JAK3*) responsible for T cell migration and activation compared to regions with normal hair growth in alopecia areata patients (Table 2) (136).

Genome-wide association studies (GWAS) were performed to further investigate how innate and adaptive immunity is involved in the pathogenesis of alopecia areata. Several susceptibility loci for alopecia areata were identified: *CTLA-4, IL-2, IL-2RA, HLA, ULBP*, and *Eos* (Table 2) (124). These genes are known to regulate T cell activation and proliferation. Both CTLA-4 and IL-2RA are critical regulators of regulatory T cells (124, 138). ULBP functions as a NKG2D ligand and is a stress signal, which activates γδ T cells, natural killer T cells, and CD8^+^ T cells (124, 139). Murine studies further implicate CD8^+^NK2GD^+^ T cells in the induction of alopecia areata (140). Skin of alopecia-prone C3H/HeJ mice revealed an increased number of CD8^+^NK2GD^+^ T cells with smaller numbers of CD4^+^ T cells, and mast cells (140–142). Similar to the GWAS studies, microarray analysis identified an upregulation of both IL-2 and IL-15 in skin from C3H/HeJ mice (140). IL-2 and IL-15 regulate the production of IFN-γ by CD8^+^ effector T cells and NK cells which induces a positive loop, promoting a type I cellular immune response and inflammation in the hair follicle (140, 143, 144). More recent meta-analysis studies on alopecia areata have further identified candidates that regulate autophagy/apoptosis, T regulatory cells and the JAK/STAT pathway (145). Patients with alopecia areata have elevated JAK3 protein levels in the epidermis and phosphorylated JAK3 in the dermal infiltrate (146).

Together, these findings have led to the investigation of several treatments that help regrow hair in patients with alopecia areata (147). However, none of the treatments have been approved by the FDA due to severe study limitations and lack of pediatric treatment studies on alopecia areata (147). Corticosteroids may be administered orally, topically, or intralesionally but recurrence is likely. Clinical studies have focused on the delivery and timing of corticosteroid treatments to improve results for hair regrowth (148–150). Beyond corticosteroids, researchers have begun to study the effectiveness of JAK inhibitors. Ruxolitinib is a JAK1/2 inhibitor that blocks IFN-γ signaling, which is normally utilized by CD8^+^NKG2D^+^ lymphocytes (140). Ruxolitinib is currently approved by the FDA for the treatment of polycythemia vera and myelofibrosis; however, it is currently being studied in alopecia areata patients. In 2016, ruxolitinib was administered orally to 12 patients 2× daily with 20 mg for 3–6 month and 75% of the patients exhibited an average hair regrowth of 92% (151). Baricitinib, another immunomodulator that inhibits JAK1/2 is being studied in clinical trials for alopecia areata (152). A trial starting in 2012 enrolled one patient, and in 9 months that patient completely sustained hair regrowth (152). Tofacitinib is a small molecule JAK3 inhibitor. In an open-label pilot study, tofacitinib was administered to 12 patients, 8 patients exhibited more than 50% hair regrowth, 3 patients demonstrated less than 50% hair regrowth, while 1 patient did not demonstrate hair regrowth (153). Ongoing studies are further investigating how modulating cytokine signaling *via* the JAK/STAT pathway in T lymphocytes can effectively treat alopecia areata.

## Diabetes Mellitus Type II

Diabetes mellitus is a chronic condition of elevated blood glucose levels caused by insufficient insulin production and/or insulin resistance. Approximately 30.3 million people in the US have been diagnosed with diabetes, with 90–95% of these cases being diabetes mellitus type 2 (T2DM) (154). Obesity is a strong predictor for T2DM (155). Patients with T2DM exhibit a complex array of complications including a higher prevalence of chronic non-healing wounds, impaired leukocyte function, neuropathy, and vasculopathy (156, 157).

Chronic non-healing wounds in T2DM patients are caused by a disruption in one or more stages of the normal wound healing process (158, 159). The process of wound healing normally encompasses numerous overlapping stages. First there is clot formation from platelet aggregation followed by cytokine and chemokine secretion which elicits an inflammatory response. This leads to the proliferation of epithelial cells to restore the lost barrier. Finally, the tissue is remodeled to strengthen the new matrix. Skin-resident T cells participate in the early stages of wound healing through the production of growth factors, cytokines, and chemokines (7). Skin-infiltrating T cells arrive within a week to fight infection and secrete cytokines including IFN-γ (160). However, in T2DM the timing and level of immune cell function becomes altered.

Diabetic wounds become arrested in a chronic state of inflammation that is caused by pro-inflammatory cytokines, such as TNF-α, secreted by adipocytes and immune cells (161). In murine models of obesity and T2DM, epidermal γδ T cells become reduced in number as disease progresses (100). The remaining T cells in the epidermis exhibit reduced cytokine and growth factor production during wound repair (100). Growth factor production by the γδ T cells is partially restored upon blocking TNF-α with antibodies prior to injury (100). T cell dysfunction in obese, T2DM mice results in a thinner epidermis with premature keratinocyte differentiation (85). In accordance, the blockade of TNF-α improves insulin resistance in animal models (162), but has not shown good efficacy in humans (163).

T cells isolated from patients with chronic non-healing wounds lack the ability to secrete growth factors such as IGF-1 upon stimulation (7). This suggests that skin T cells become refractory to stimulation as wound healing stalls. Obese subjects also exhibit a reduction in Vγ2Vδ2 T cells in the blood that negatively correlates with BMI (65). The remaining Vγ2Vδ2 T cells are less able to become activated and secrete reduced levels of IFN-γ in response to virus infected cells (65). The Vγ2Vδ2 T cells in obese subjects show an increase in differentiation from central memory to T effector memory T cells and T effector memory CD45RA^+^ lymphocytes (65). Future studies are needed to further elucidate the contribution of T cell dysregulation in obese T2DM patients to chronic non-healing ulcers (164).

Conventionally, diabetic foot ulcers are treated with wound debridement followed by aseptic techniques that aim to keep the area clean and moist (165). Medications that reduce inflammation directly and/or indirectly, such as metformin, are being studied to determine whether they improve diabetic wound healing (166, 167). Upon activation of AMP-activated protein kinase, metformin suppresses the production of glucose by the liver (168). The activation of AMPK results in anti-inflammatory responses through the suppression of NF-κβ signaling (169). Metformin reduces Th17 differentiation and IL-22 secretion, decreasing chronic inflammation and improving immune responses (170, 171).

Systemic insulin therapy has shown some success in improving wound healing in rats and humans (156, 172). Insulin regulates glucose uptake, gene expression, and cell differentiation which all impact skin-resident T cells and wound repair. Other studies show that topical insulin injections accelerate the healing of diabetic foot ulcers by stimulating the AKT and ERK pathways (156). A large multicenter clinical trial is needed to determine the level of effectiveness and mechanisms used by insulin to promote proper T cell function and wound healing in diabetic patients.

## Melanoma

Melanoma only makes up approximately 1% of skin cancer cases, yet it is responsible for most skin cancer deaths with an estimated 9,730 deaths and 87,110 new cases in 2017 (173, 174). The majority of melanomas are caused by UV radiation from sun exposure (175). Malignant melanoma has a high metastatic rate, making it one of the most aggressive and dangerous cancers (176). Cytotoxic T cells recognize antigens associated with melanoma, including tyrosinase and tyrosinase-related proteins 1 and 2; however, the immune response is inhibited or repressed by the tumor environment (176–179).

There is a reduction in number and percentage of circulating Vγ2Vδ2 T cells in melanoma patients (180). Interestingly, 15–25% of tumor-infiltrating cells in patients are Vγ2Vδ2 T cells (180). γδ T cell cytotoxicity in melanoma patients is also significantly lower compared to healthy patients and this reduction in cytotoxicity is correlated with melanoma stages (180). In a study of 46 patients, 23 of the patients’ melanoma-infiltrating γδ T cells into the skin were Vδ1+ T cells, 19 patients had predominantly Vδ2+, and 4 patients did not have a significant different in percentages of Vδ1^+^ and Vδ2^+^ T cells (60). Cytotoxic capability between Vδ1^+^ and Vδ2^+^ among these patients was substantially different. Most Vδ1^+^ T cells performed cytotoxic activity against the melanoma cell line A375; however, only two out of eight Vδ2^+^ T cells exhibited this capability (60). Thus, γδ T cell subsets play complex roles that contribute to immunosurveillance of melanoma in human skin.

**Figure 2:**
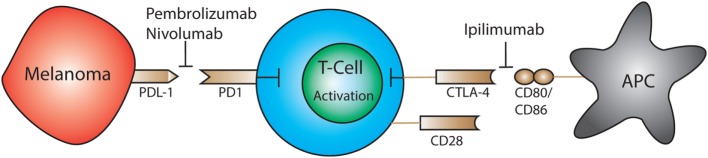
Expression of PD-1 and CTLA-4 by T cells leads to downregulation of activation and antitumor cytotoxic activity by suppressing downstream TCR signals. Immunotherapeutics have been approved that block CTLA-4 (ipilimumab) or programmed cell death protein 1 (PD1) (pembrolizumab, nivolumab) which restores the ability of T cells to become activated and destroy tumor cells.

Melanoma utilizes a wide variety of mechanisms to evade the host’s immune system. The most recent mechanisms under extensive investigation are CTLA-4, programmed cell death protein 1 (PD1), and programmed death-ligand 1 (PDL1) immune checkpoints (Figure 2) (102). T cell expression of CTLA-4 and PD1 downregulates functions such as activation and antitumor activity by suppressing signals downstream of TCR stimulation (177, 178). CTLA-4 binds to CD80 and CD86 expressed by antigen-presenting cells. CTLA-4 disrupts CD80/CD86- CD28 binding, which suppresses co-stimulation and T cell activation (181). PD1 is a transmembrane protein that is expressed on activated T cells, DCs, B cells, and NK cells. It binds to the ligands PDL1 and PDL2 resulting in the suppression of T cell activation (182). Resting Vγ2Vδ2 T cells in the blood express PD1 at low levels but upon activation it is upregulated (183). PD1 regulated TCR-mediated activation thus maintains self-tolerance and prevents autoimmunity (184). Cancer cells express PDL1 and as a result bypass immune checkpoints and evade T cell recognition (181). Although anti-CTLA-4 and anti-PD1 treatments have shown promise, it is necessary to further investigate how these treatments impact Vδ1^+^ T cells specifically.

The FDA-approved drug, ipilimumab, targets CTLA-4 on T cells for the treatment of melanoma. This medication is also used to treat autoimmune diseases such as multiple sclerosis and rheumatoid arthritis (185). Targeting CTLA-4 reduces Treg-mediated suppression and enables activation and proliferation of T cells (186–188). Beyond ipilimumab, the FDA has also approved immunotherapeutics that target the PD1 pathway: pembrolizumab and nivolumab. By blocking the PD1 receptor the therapeutic antibody prevents ligation with PDL1, which is expressed on tumor cells; this restores the ability of T cells to respond to melanoma antigens and initiate cytotoxic responses and cytokine production (189).

A phase III study of 945 patients with stage III and IV melanoma across 137 countries revealed that when combined, ipilimumab and nivolumab have synergistic effects against metastatic melanoma (185). Unfortunately, these patients also experienced side effects from the combined treatment including diarrhea, elevate liver enzymes, and colitis (185). This suggests that while a combination of CTLA-4 and PD1 targeted therapies can be more successful than a monotherapy, it is also likely to cause adverse effects by unregulated T cells.

## Conclusion

αβ and γδ T cells are vital in the maintenance and homeostasis of the skin through the recognition of stressed or damaged cells and subsequent functions including the secretion of cytokines and growth factors. Current studies do not allow precise conclusions on the distinct roles of αβ and γδ in skin immunity to be drawn. However, both αβ and γδ T cells fight pathogens and cancer by directly destroying infected or transformed cells in the skin, while also maintaining immunological tolerance. γδ T cells recognize a wide variety of peptide and non-peptide antigens released by stressed, damaged, malignant, or infected cells in the skin while αβ T cells recognize peptides derived from pathogens or tumors presented by MHC. Through specific cytokine profiles CD4^+^ αβ T cells aid in the recruitment and regulation of other immune cells, while CD8^+^ αβ T cells exhibit cytotoxicity (190–192). The receptors responsible for T cell activation also differ between αβ and γδ T cells, suggesting unique roles in skin homeostasis and immunity. In addition, skin-resident versus recirculating T cells show distinct profiles indicating that the various roles and requirements for T cell function in the skin is complex and requires subsetting. Thus, the molecular mechanisms that regulate skin-specific αβ and γδ T cells are important to elucidate for the development and study of immunotherapies.

Cytokine and growth factor production by αβ and γδ T cells helps to maintain skin homeostasis, while factors produced upon activation contribute to wound repair and the eradication of tumors. Elevated and unregulated T cell activity can contribute to or cause chronic inflammatory related diseases including psoriasis, and alopecia areata. Conversely, defects in T cell function can increase susceptibility to melanoma. Current knowledge about skin αβ and γδ T cell activation and antitumor activity has advanced considerably, yet further studies are needed to identify specific molecular mechanisms that can be exploited for therapeutics that treat autoimmune diseases and cancer.

## Author Contributions

MC, AD, AR, and JJ wrote the manuscript, provided critical discussion in manuscript preparation, and revised the manuscript.

## Conflict of Interest Statement

The authors declare that the research was conducted in the absence of any commercial or financial relationships that could be construed as a potential conflict of interest.

## Abbreviations

DC, dendritic cells; ILCs, innate lymphoid cells; DN, double negative; DETC, dendritic epidermal T cells; LAT, linker activation in T cell; CLA-1, cutaneous lymphocyte antigen-1; ICAM-1, intercellular adhesion molecule 1; VCAM-1, vascular cell adhesion protein 1; MICA, MHC class I related chain A molecules; MICB, MHC class I related chain B molecules; AICD, activation induced cell death; JAML, junctional adhesion molecule-like; B-CLL, lymphocytic leukemia of B-cell type; JAK3, janus kinase 3; T2DM, diabetes mellitus type 2; PD1, programmed cell death protein 1; PDL1, programmed death-ligand 1; LFA-1, lymphocyte function-associated antigen 1.
